# CAN OLDER INPATIENTS WITH MOBILITY LIMITATION BENEFIT FROM MULTIDISCIPLINARY INTERVENTIONS: M-MOBILE STUDY

**DOI:** 10.1093/geroni/igae098.1211

**Published:** 2024-12-31

**Authors:** Lina Ma

**Affiliations:** Xuanwu Hospital, Capital Medical University, Beijing, Beijing, China (People’s Republic)

## Abstract

**Objective:**

Mobility limitation, the loss of exercise capacity or independent living ability, is a common geriatric syndrome. As a reversible precursor to disability, it is crucial to comprehensively assess the factors of mobility limitation and administer individualized multidisciplinary team (MDT) treatment. We hypothesized that MDT can improve mobility in older adults with mobility limitation methods: We conducted a study titled “A Multidisciplinary-team approach for management of Mobility Limitation in Elderly (M-MobiLE)”, which is a multicenter, randomized, and controlled trial. A total of 442 older inpatients with mobility limitation from five hospitals were recruited. Mobility limitation was defined as Short-Physical Performance Battery (SPPB) ≤9. Activities of Daily Living (ADL) and intrinsic capacity were assessed. The intervention group received an exercise-centered individualized MDT treatment, including exercise, educational, nutritional, and medical interventions; while the control group received routine medical treatment. All patients were followed up for six months. Adverse events such as falls, fracture, stroke, cardiovascular events and death were recorded.

**Results:**

Preliminary data analysis showed that compared to the routine medical treatment, the exercise-centered individualized MDT treatment reduces adverse events at 6 months (6.3% vs 13.0%, p=0.036). However, there was no statistical difference in ADL, length of stay, and hospital costs between the two groups at 6 months after discharge.

**Conclusion:**

This study developed a multidisciplinary decision-making clinical pathway for older inpatients with mobility limitation, which had potential clinical benefits such as reducing short-term adverse events in older inpatients with mobility limitation in preliminary data analysis. Further analysis is ongoing.

